# Proteomics and Network Analyses Reveal Inhibition of Akt‐mTOR Signaling in CD4^+^ T Cells by *Mycobacterium tuberculosis* Mannose‐Capped Lipoarabinomannan

**DOI:** 10.1002/pmic.201700233

**Published:** 2017-11-21

**Authors:** Ahmad F. Karim, Obondo J. Sande, Sara E. Tomechko, Xuedong Ding, Ming Li, Sean Maxwell, Rob M. Ewing, Clifford V. Harding, Roxana E. Rojas, Mark R. Chance, W. Henry Boom

**Affiliations:** ^1^ Department of Medicine University Hospitals Cleveland Medical Center Case Western Reserve University Cleveland OH USA; ^2^ Department of Molecular Biology & Microbiology Case Western Reserve University Cleveland OH USA; ^3^ Department of Pathology University Hospitals Cleveland Medical Center Case Western Reserve University Cleveland OH USA; ^4^ Center for Proteomics & Bioinformatics Case Western Reserve University Cleveland OH USA; ^5^ Centre for Biological Sciences University of Southampton Southampton UK; ^6^ Department of Nutrition School of Medicine Case Western Reserve University Cleveland OH USA

**Keywords:** Akt, CD4^+^ T‐cell, label‐free mass spectrophotometry, *M. tuberculosis*, ManLAM, mTOR

## Abstract

*Mycobacterium tuberculosis* (Mtb) cell wall glycolipid mannose‐capped lipoarabinomannan (ManLAM) inhibits CD4^+^ T‐cell activation by inhibiting proximal T‐cell receptor (TCR) signaling when activated by anti‐CD3. To understand the impact of ManLAM on CD4^+^ T‐cell function when both the TCR–CD3 complex and major costimulator CD28 are engaged, we performed label‐free quantitative MS and network analysis. Mixed‐effect model analysis of peptide intensity identified 149 unique peptides representing 131 proteins that were differentially regulated by ManLAM in anti‐CD3‐ and anti‐CD28‐activated CD4^+^ T cells. Crosstalker, a novel network analysis tool identified dysregulated translation, TCA cycle, and RNA metabolism network modules. PCNA, Akt, mTOR, and UBC were found to be bridge node proteins connecting these modules of dysregulated proteins. Altered PCNA expression and cell cycle analysis showed arrest at the G2M phase. Western blot confirmed that ManLAM inhibited Akt and mTOR phosphorylation, and decreased expression of deubiquitinating enzymes Usp9x and Otub1. Decreased NF‐κB phosphorylation suggested interference with CD28 signaling through inhibition of the Usp9x‐Akt‐mTOR pathway. Thus, ManLAM induced global changes in the CD4^+^ T‐cell proteome by affecting Akt‐mTOR signaling, resulting in broad functional impairment of CD4^+^ T‐cell activation beyond inhibition of proximal TCR–CD3 signaling.

## 1 Introduction

CD4^+^ T cells have a central role in controlling *Mycobacterium tuberculosis* (Mtb) infection.^1^ Despite immune control, Mtb persists by interfering with macrophage and T‐cell function, allowing for pathogen survival. We demonstrated direct and indirect inhibition of CD4^+^ T‐cell activation by different Mtb molecules, including lipoproteins LpqH, LprA, and LprG, and more recently glycolipid mannose‐capped lipoarabinomannan (ManLAM).^2^, ^3^, ^4^, ^5^ ManLAM is abundant in the Mtb cell wall, found in membrane vesicles produced by Mtb, in Mtb granulomas, and most recently in CD4^+^ T cells from lungs of Mtb‐infected mice.^6^, ^7^ ManLAM interferes with T‐cell receptor (TCR) proximal signaling by downregulating phosphorylation of Lck, CD3ζ, ZAP70, and LAT, and can induce T‐cell anergy, and thus potentially a major modulator of host T cells response to Mtb.^8^, ^9^

**Significance of the Study**

Incomplete understanding of Mtb's immune evasion mechanisms is a major barrier to development of improved TB vaccines and optimizing treatment. CD4^+^ T cells have a central role in controlling Mtb. Despite immune control, Mtb persists by interfering with macrophage and T‐cell function, allowing pathogen survival. We have demonstrated direct and indirect inhibition of CD4^+^ T‐cell activation by different Mtb molecules including lipoproteins LpqH, LprA and LprG, and glycolipid ManLAM. ManLAM is abundant in the Mtb cell wall and interferes with TCR signaling by downregulating phosphorylation of Lck, CD3ζ, ZAP70, and LAT. In this study, we show that ManLAM inhibits the Akt‐mTOR pathway, an immune signaling pathway important for productive CD4^+^ T‐cell function. Understanding the role of ManLAM in Mtb's immune evasion mechanisms is not only essential for understanding Mtb's interaction with the host's immune system, but also for new approaches to TB vaccine development and host‐directed therapies.

T‐cell activation through the TCR–CD3 complex leads to marked changes in the proteome of T cells. Optimal T‐cell activation requires coordinated signaling through the main costimulatory molecule CD28 (signal 2) at the same time as TCR (signal 1) interacts with MHC + peptide, and later through the interaction of IL‐2 with IL‐2R. These coordinated signaling pathways allow CD4^+^ T cells to enter into the cell cycle, produce cytokines, and proliferate and differentiate from naïve to effector and memory T cells. These processes require coordination of multiple signaling pathways activated through TCR–CD3, CD28 and IL‐2R.^10^ Earlier studies focused on early signaling events through TCR–CD3 only. This study aimed to determine downstream mechanisms and major signaling pathways affected by ManLAM responsible for the inhibition of proliferation, IL‐2, and IFN‐γ production, and more recently induction of anergy.^11^ Specifically, we wanted to determine if ManLAM affected CD28 signaling and function.

MS has characterized TCR complex formation^12^, ^13^, ^14^, ^15^, ^16^ and the effect of a range of stressors on the T‐cell proteome.^17^, ^18^, ^19^ Recent advances have overcome technical issues in quantitative MS and allow analysis of the complexity and dynamic range of the cellular proteome.^20^ To extract biological meaning, different bioinformatics tools have been developed to assist in interpreting MS‐based cellular studies.^21^, ^22^

In this study, we used label‐free quantitative MS to characterize the effect of ManLAM on the CD4^+^ T‐cell proteome when these cells are activated through both the TCR‐CD3 complex and CD28. Approximately 5000 peptides were identified and quantified from three biological experimental datasets in primary murine CD4^+^ T cells activated with anti‐CD3 and anti‐CD28 mAbs in the presence or absence of ManLAM. Peptides with varying abundance were selected by likelihood ratio based statistical significance by comparing the intensities under different experimental conditions. ManLAM treatment resulted in significant changes in the abundance of 149 peptides representing 131 proteins in the activated CD4^+^ T‐cell proteome that affected a variety of enriched cluster modules and pathways. Validation experiments confirmed ManLAM‐induced inhibition of proliferating cell nuclear antigen (PCNA) that regulates cell cycle progression. Protein–protein interaction (PPI) network analysis revealed a central role for the Akt/mammalian target of rapamycin (mTOR) signaling pathway and ubiquitination processes. Western blot (WB) studies revealed decreased phosphorylation of both Akt and mTOR in activated CD4^+^ T cells exposed to ManLAM, as well as decreased expression of deubiquitinases Otub1 (ovarian tumor deubiquitinase ubiquitin aldehyde binding 1) and Usp9x (ubiquitin‐specific peptidase 9, X‐linked), key molecules that regulate mTOR‐mediated master transcription factor NF‐κB (nuclear factor kappa light‐chain‐enhancer of activated B cells). Thus, disruption of upstream TCR, CD28, and IL‐2R signaling by ManLAM in the activated CD4^+^ T‐cell proteome is mediated downstream primarily through inhibition of Akt‐mTOR signaling.

## 2 Experimental Section

### 2.1 Mice

Females C57BL/6J aged 8–10 weeks were purchased from Charles River Laboratories (Wilmington, MA). Mice were housed under specific pathogen‐free conditions. All experiments were performed in compliance with the U.S. Department of Health and Human Services Guide for the care and use of Laboratory Animals and were approved by the Institutional Animal care and Use Committee at Case Western Reserve University (protocol number: 2015‐0030).

### 2.2 Isolation and Activation of CD4^+^ T Cells

Murine CD4^+^ T cells were isolated from spleens of 8‐ to 10‐week‐old mice. Tissues were dissociated, and red blood cells lysed in hypotonic lysis buffer (10 mM Tris‐HCl and 0.83% ammonium chloride). Splenocytes were plated in 100‐mm tissue culture plates and allowed to adhere for 1 h at 37 °C. Untouched CD4^+^ T cells were purified from nonadherent splenocytes using a CD4^+^ T‐cell‐negative isolation kit (Miltenyi Biotec) by following manufacturer's instructions (purity > 97%). CD4^+^ T cells were rested overnight in complete DMEM (BioWhittaker, East Rutherford, NJ) supplemented with 0.5 mM 2‐mercaptotoethanol, 10 mM HEPES buffer, nonessential amino acids, 2 mM L‐glutamine, penicillin/streptomycin, and 10% fetal bovine serum prior to use in assays. Prior to activation, CD4^+^ T cells were pretreated with or without purified ManLAM (40 μg/mL) for 1 h at 37 °C in a 5% CO_2_ incubator. ManLAM from Mtb H37Rv was obtained from the Tuberculosis Vaccine Testing and Research Materials contract (NIAID HHSN266200400091C) at Colorado State University (CSU). ManLAM or mock pretreated CD4^+^ T cells were washed and excess ManLAM removed before stimulation with anti‐CD3 and anti‐CD28 mAbs. Stimulation of CD4^+^ T cells was performed in serum‐free HL‐1 medium (BioWhittaker) supplemented with 0.5 mM 2‐mercaptoethanol, 10 mM HEPES buffer, nonessential amino acids, 2 mM l‐glutamine, and penicillin/streptomycin. CD4^+^ T cells (2 × 10^6^ cells/well) were activated in 6‐well plates with 1 μg/mL of soluble antimouse CD28 (clone 37.51) in wells precoated with 1 μg/mL of hamster antimouse CD3ε (145‐2C11) mAb (both from BD Biosciences) as previously described.^8^, ^9^ After 24 h cells were harvested and lysed for MS analysis. Supernatants were collected for IL‐2 measurements to confirm that ManLAM had inhibited CD4^+^ T‐cell activation.^8^, ^9^ For experiments with rapamycin (10 nM; Cell Signaling Technology), T cells were pretreated as described for ManLAM.

### 2.3 Sample Preparation for Solution‐Based Label‐Free Proteomics

Samples were prepared and digested using a protocol adapted from the filter‐aided‐based sample preparation method.^23^ Each sample was solubilized and lysed in 5% SDS and 0.1 M DTT in 0.1 M Tris HCL pH 7.6. Clarified samples were processed for SDS detergent removal, and reduction and alkylation using the filter‐assisted sample preparation method. Total protein concentration was determined using a modified Bradford assay (Bio‐Rad Laboratories). Each sample was adjusted for equal amounts of protein in 13 μL with 50 mM Tris pH 8.0. Dithiothreitol was added to a final concentration of 5 mM and samples were reduced at 37 °C for 30 min and cooled to room temperature prior to alkylation with iodacetamide at a final concentration of 10 mM for 30 min. A dual proteolytic digestion (1:1 ratio) was performed with endopeptidase Lys C (Wako Chemicals, Richmond, VA) and trypsin (Pierce, Thermo Fisher Scientific, Waltham, MA) with a final enzyme to protein ratio of 1/20 (w/w). First, Lys C was added and incubated for 2 h at 37 °C and then adjusted to 2 M urea with 50 mM Tris pH 8.0 to accommodate the trypsin digestion that incubated overnight at 37 °C.

### 2.4 Experimental Design

Samples from three independent experiments yielding 500–700 ng of protein were run. Each sample was analyzed in triplicate, resulting in 36 analytical runs (three biological replicates × four experimental conditions × three technical replicates). Repeat analyses of sample material were performed to improve protein identification. Detailed experimental groups and technical replicates are shown in Table 1 with the designated treatment for each sample. MS data for each sample are stated in Table S1, Supporting Information.

**Table 1:** Details of the experimental groups and designated treatments for the Mass spec samples used for statistical analysis

Sample	Experimental Groups		Treatments
**500ng **			
Intensity [53 – SW_1G]	CD4^+^ T cells	Technical Replicates	
Intensity [69 – SW_1H]	CD4^+^ T cells		Control
Intensity [63 – SW_1I]	CD4^+^ T cells		
Intensity [67 – SW_2G]	CD4^+^ T cells + αCD3/ αCD28	Technical Replicates	
Intensity [61 – SW_2H]	CD4^+^ T cells + αCD3/ αCD28		Treatment 1
Intensity [49 – SW_2I]	CD4^+^ T cells + αCD3/ αCD28		
Intensity [55 – SW_3G]	CD4^+^ T cells + αCD3/ αCD28 + LAM	Technical Replicates	
Intensity [51 – SW_3H]	CD4^+^ T cells + αCD3/ αCD28 + LAM		Treatment 2
Intensity [59 – SW_3I]	CD4^+^ T cells + αCD3/ αCD28 + LAM		
Intensity [65 – SW_4G]	CD4^+^ T cells + LAM	Technical Replicates	
Intensity [57 – SW_4H]	CD4^+^ T cells + LAM		Treatment 3
Intensity [71 – SW_4I]	CD4^+^ T cells + LAM		
**600ng**			
Intensity [1 – SW_1D]	CD4^+^ T cells	Technical Replicates	
Intensity [5 – SW_1E]	CD4^+^ T cells		Control
Intensity [41 – SW_1F]	CD4^+^ T cells		
Intensity [29 – SW_2D]	CD4^+^ T cells + αCD3/ αCD28	Technical Replicates	
Intensity [3 – SW_2E]	CD4^+^ T cells + αCD3/ αCD28		Treatment 1
Intensity [11 – SW_2F]	CD4^+^ T cells + αCD3/ αCD28		
Intensity [47 – SW_3D]	CD4^+^ T cells + αCD3/ αCD28 + LAM	Technical Replicates	
Intensity [25 – SW_3E]	CD4^+^ T cells + αCD3/ αCD28 + LAM		Treatment 2
Intensity [13 – SW_3F]	CD4^+^ T cells + αCD3/ αCD28 + LAM		
Intensity [9 – SW_4D]	CD4^+^ T cells + LAM	Technical Replicates	
Intensity [7 – SW_4E]	CD4^+^ T cells + LAM		Treatment 3
Intensity [35 – SW_4F]	CD4^+^ T cells + LAM		
**700ng**			
Intensity [19 – SW_1A]	CD4^+^ T cells	Technical Replicates	
Intensity [37 – SW_1B]	CD4^+^ T cells		Control
Intensity [27 – SW_1C]	CD4^+^ T cells		
Intensity [15 – SW_2A]	CD4^+^ T cells + αCD3/ αCD28	Technical Replicates	
Intensity [45 – SW_2B]	CD4^+^ T cells + αCD3/ αCD28		Treatment 1
Intensity [21 – SW_2C]	CD4^+^ T cells + αCD3/ αCD28		
Intensity [23 – SW_3A]	CD4^+^ T cells + αCD3/ αCD28 + LAM	Technical Replicates	
Intensity [43 – SW_3B]	CD4^+^ T cells + αCD3/ αCD28 + LAM		Treatment 2
Intensity [31 – SW_3C]	CD4^+^ T cells + αCD3/ αCD28 + LAM		
Intensity [33 – SW_4A]	CD4^+^ T cells + LAM	Technical Replicates	
Intensity [39 – SW_4B]	CD4^+^ T cells + LAM		Treatment 3
Intensity [17 – SW_4C]	CD4^+^ T cells + LAM		

### 2.5 Liquid Chromatography and Mass Spectrometry for Discovery

LC‐MS/MS was performed using a Waters ultra‐high‐pressure LC NanoAcquity (Waters Corporation, Milford, MA), an LTQ Orbitrap Velos and an Orbitrap Elite (Thermo Fisher Scientific). The order of sample injections was randomized across all samples. The instrument was mass calibrated immediately before analysis using the instrument protocol. Mobile phase A (aqueous) contained 0.1% formic acid in 5% ACN and mobile phase B (organic) contained 0.1% formic acid in 85% ACN. Samples were trapped and desalted online in mobile phase A at 10 μL/min for 10 min using a Waters UPLC PST C18 nanoACQUITY 300 (75 μm × 25 cm) reversed‐phase column with 5% mobile phase B. The column was washed at 99% mobile phase B for 10 min, followed by reequilibration at 100% A for 15 min. Positive mode electrospray was conducted using a nanospray source and the mass spectrometer was operated at a resolution of 60 000. Quantitative and qualitative data were acquired using alternating full MS scan and MS/MS scans in normal mode. Survey data were acquired from *m*/*z* of 400–1600 and up to 20 precursors based on intensity were interrogated by MS/MS per switch. Two microscans were acquired for every precursor interrogated and MS/MS was acquired as centroid data. All MS analytical parameters for the discovery samples of the LC/MS/MS analytical run time was extended to 240 min (4 h). The whole dataset was processed via Rosetta Elucidator 11 (version 3.3.01 SP4 25) (Rosetta Biosoftware, Seattle, WA). The MS/MS peak lists were subsequently searched by MASCOT (version 2.2.0, IPI_mouse_06_2010) (Matrix Science, London, UK). The database used was mouse International Protein Index (IPI) (56 957 sequences). Corresponding IPI identifiers were mapped to UniProt using the last version of UniProt to support the mapping (2014_01). MS search settings were as follows: trypsin enzyme specificity; mass accuracy window for precursor ion, 10 ppm; mass accuracy window for fragment ions, 0.8 Da; variable modifications including carbamidomethlylation of cysteines, one missed cleavage, and oxidation of methionine. To provide additional confidence in the assignments, we considered proteins that had ≥ 2 peptides matching the above criteria to be confirmed assignments while proteins identified with one peptide with the above criteria as tentative assignments. Additionally, false discovery rate (FDR) of 2% was determined following the assignment of peptides and proteins using tellers employed within Rosetta Elucidator (Rosetta Biosoftware).^24^ Peptides that passed the identification threshold were assigned for automated differential quantification and reported.

LC‐MS/MS raw data were imported and for each MS spectrum profile of each LC‐MS/MS run, chromatographic peaks and monoisotopic masses were extracted and aligned. Peak lists with the monoisotopic mass and corresponding MS/MS data were then generated for each sample and searched using MASCOT. Resultant peptide identifications were imported into Elucidator and monoisotopic masses annotated with peptide identifications. Average peptide intensities from the same protein from triplicate samples were summed together to provide fold changes and log transformed with respect to their control groups for each protein.

The identified peptide level data were exported from MASCOT to Excel spreadsheet files for further data analysis (three datasets added as Supporting Information file). All the MS proteomics raw data have been deposited to the ProteomeXchange Consortium via the PRIDE partner repository with the dataset identifier PXD004164, https://doi.org/10.6019/PXD004164.

### 2.6 Statistical Rationale

Prior to analysis, data quality/reproducibility was addressed by intra‐class correlation coefficient (ICC). The results showed that the median value of ICC was greater than 0.72, which indicated that data reproducibility was good overall and could proceed for further detailed analysis.

The rate ratio (RR) of average value of one treatment group versus average value of another treatment group was calculated. There were six RRs designated to different experimental groupings: control versus treatment 1 (experimental Group 1), control versus treatment 2 (experimental Group 2), control versus treatment 3 (experimental Group 3), treatment 1 versus treatment 2 (experimental Group 4), treatment 1 versus treatment 3 (experimental Group 5), and treatment 2 versus treatment 3 (experimental Group 6) (see table above for details). (Note, for easy coding the treatments were renamed, i.e., control = resting CD4^+^ T cells [unstimulated]; treatment 1 = resting CD4^+^ T cells + antiCD3/CD28 [activated CD4^+^ T cells]; treatment 2 = resting CD4^+^ T cells + antiCD3/CD28 + ManLAM [activated CD4^+^ T cells + ManLAM]; treatment = resting CD4^+^ T cells + ManLAM [unstimulated + ManLAM].)

Selection of “winner” peptides was based on expression differences between treatment groups (there are six pairwise comparisons). Likelihood ratio test based on mixed‐effect model was applied to correctly handle the correlation structure of data that had three technical measurements for each biological sample.^25^ The FDR controlling procedure was adapted to handle multiple comparison issues when testing many peptides simultaneously.^26^ The final peptides were based on an FDR‐adjusted *p*‐value cut‐off 0.1. Since the peptide search was in the “exploratory” stage, we used FDR adjustment but set a “relaxed” threshold of 0.1 to capture a sufficient number of potential candidates for downstream analysis and validation. In this study and subsequent analyses, the primary focus was on the effect of ManLAM on activated CD4^+^ T cells, that is, Group 4. RR, *p* values, and FDR‐adjusted *p* values were saved for all four pairs of comparisons and listed in Table S2 (A–D), Supporting Information.

### 2.7 Construction of Proteome Functional Networks and Pathway Analysis

Following statistical analysis, winner peptides were listed as differentially regulated proteins and imported into Crosstalker (YourOmics, Inc. www.youromics.com). The Crosstalker method filters and augments an input set of proteins in the context of molecular interaction networks to remove input proteins that show little association with the input set, and to recommend novel proteins (noninput proteins present in the network) that show significantly greater than random association with the input set. An abbreviated technical description follows below, and a recent publication provides greater technical details and links to a publicly available implementation.^27^ Once imported, the software utilizes biomedical literature and protein interaction databases such as STRING 10 (Search Tool for the Retrieval of Interacting Genes/Proteins)^28^ to elucidate biological networks and pathways within the uploaded protein lists. To identify functionally enriched modules, Crosstalker uses the method of Nibbe et al.^29^ to identify proteins with a low likelihood of being in proximity to the seed proteins by chance, that is, proteins that directly or indirectly interact more with the set of seed proteins than they interact with the majority of other similar sets of proteins in the network. Crosstalker first estimates the proximity of all proteins in a selected molecular interaction network to the seed proteins using a random walk with restarts based method, where proteins in proximity to the seeds are expected to be functionally related under the “guilt by association” principle of PPI networks.^30^ Crosstalker then estimates a null proximity score distribution for each protein in the network using 10^4^ Monte Carlo simulations with sets of randomly sampled seed proteins of a similar degree distribution to the experimental seed proteins. Finally, the proximity to the true seeds of every protein in the network is tested against its corresponding null distribution to identify node scores significant at a *p* < 0.001 level, that is, “Crosstalker” nodes. Note that Crosstalker nodes can be seed proteins or non‐seed proteins, as the statistical test is performed on all proteins in the network, and this can both remove seed proteins from results as well as add novel proteins. Network modules are then assembled by inducing connected components from the set of all Crosstalker nodes, and the proteins in each network module are subsequently tested for pathway overrepresentation using a one‐tailed Fisher's exact test.

Some proteins that did not pass the selection criteria are included in the result visualization as “Bridge” nodes if they directly connect two or more Crosstalker nodes in different result networks, but bridge nodes do not participate in the enrichment tests. The resulting networks and enrichments are simultaneously visualized in a web‐based user interface.^27^ The network and pathway results are also disseminated using a novel workspace sharing strategy, readers of this paper can click on a hot link to access the Crosstalker network workspace and manipulate the visualizations of pathways and networks of interest. Crosstalker is freely available for noncommercial use at www.youromics.com.

### 2.8 Immunoblot Analysis

The following antibodies from Cell Signaling Technology were used for WB experiments unless otherwise mentioned: anti‐p‐Akt (Ser473) (#9271) for p‐Akt Ser 473; anti‐Akt (#9272) for total Akt; anti p‐mTOR‐ Ser2448 (D9C2) (#5536) for p‐mTOR‐Ser 2448; anti‐OTUB1(D8F7) (#3783) for Otub1; anti‐Usp9x (# 5751) for Usp9x; anti‐Phospho‐NF‐κB p65 (Ser536) (#3033) for p‐NF‐κB p65; anti‐NF‐κB p65 (C22B4) (#4764) for total NF‐κB p65 and anti‐β‐actin (#4970) for actin used as a loading control. For PCNA protein expression, we validated protein by WB with anti‐PCNA (# 8580).

Briefly, cells were harvested at indicated time points and lysates prepared from between 2 and 5 × 10^6^ cells in buffer of 20 mM Tris‐HCl, pH 7.5, 150 mM NaCl, 1 mM EDTA, 1 mM EGTA, 1% Triton X‐100, and a phosphatase inhibitor cocktail (Roche, Mannheim, Germany). Clarified supernatants were quantified for protein concentration by Bradford assay (Bio‐Rad Laboratories). Lysates were subjected to SDS‐PAGE, transferred to nitrocellulose membranes (Bio‐Rad Laboratories), and blocked with 5% BSA in PBS for 1 h at room temperature. Proteins were probed with the primary antibody, followed by HRP‐conjugated secondary antibodies (Jackson ImmunoResearch). Between antibody incubations, blots were washed with PBST (1× PBS + 0.01% tween 20) for 15–20 min (3–4 × 5 min). Proteins were visualized by chemiluminescence with ECL 2 WB substrate (Thermo Scientific). WB were analyzed with ImageJ software (NIH).

### 2.9 Intracellular and Propidium Iodide Staining

Cells were first stained with mAbs for the following surface receptors: CD3, CD4 (Biolegend). Live cell staining was performed with LIVE DEAD fixable yellow dead cell stain (eBioscience). PCNA intracellular staining was performed with anti‐PCNA conjugated with Alexa Fluor 488 (#8580). Briefly, CD4^+^ T cells were harvested and after surface staining cells were fixed with 4% paraformaldehyde for 15 min and then washed with perm wash buffer. Then cells were stained with anti‐PCNA and mouse IgG2a as isotype control for 30 min. After incubation, cells were washed with perm wash buffer, resuspended in 2% paraformaldehyde and analyzed by flow cytometry on LSRII (BD Biosciences).

For propidium iodide staining for DNA content analysis, cells were plated in 96‐well plates for 24 h for each experimental condition. After incubation, medium was aspirated and cells stained for 30 min at 4 °C with 150 μL propidium iodide staining solution containing 0.1 mg/mL propidium iodide (Sigma), 3 μL/mL Triton X‐100 (Sigma), 1 mg/mL sodium citrate (Sigma) and 20 μg/mL RNase (Sigma). Samples were analyzed by flow cytometry and analyzed with Flow‐Jo (Treestar).

## 3 Results

### 3.1 Effect of ManLAM on the Proteome of Resting and Activated Cd4^+^ T Cells

**Figure 1:**
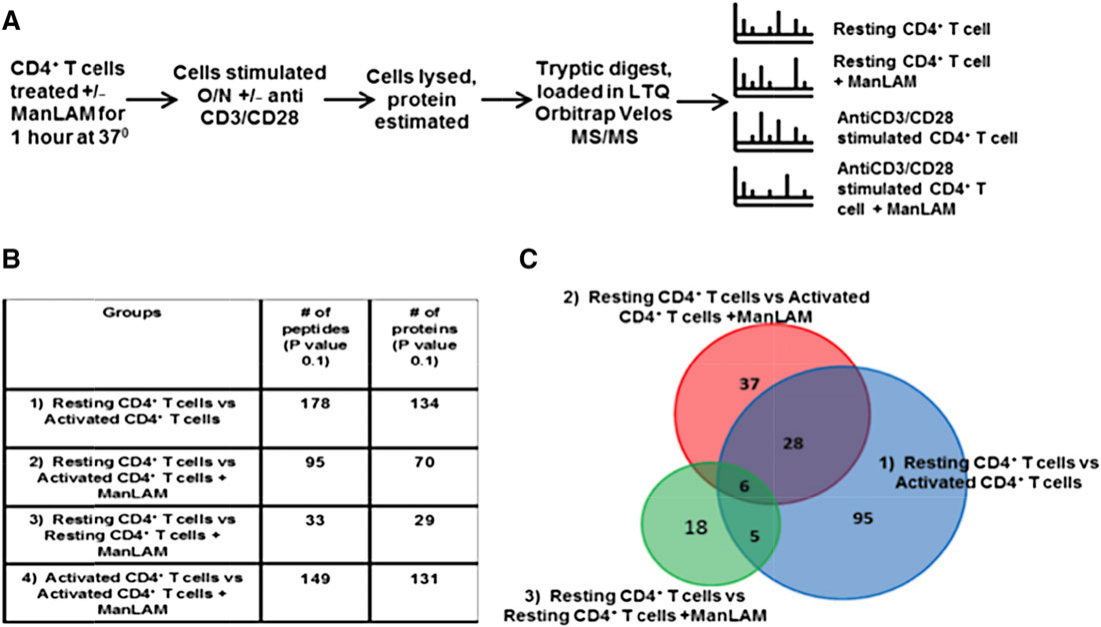
Effect of ManLAM on the proteome of resting and anti‐CD3/CD28‐activated CD4^+^ T cells. A) Experimental design for label‐free mass spectrometric analysis of the effect of ManLAM on the proteome of resting and activated CD4^+^ T cells. B) Table with total number of differentially expressed (*p* ≤ 0.1) proteins among the four experimental groups: Resting ± ManLAM CD4^+^ T cells and activated ± ManLAM CD4^+^ T cells. C) Venn diagram with the number and distribution of differentially expressed proteins in activated CD4^+^ T cells and ManLAM‐treated resting and activated CD4^+^ T cells with untreated resting CD4^+^ T cells serving as baseline.

To identify differentially expressed proteins in CD4^+^ T cells in response to ManLAM, label free MS was used to quantify peptide abundance levels across conditions of biological interest (Figure 1A). Three independent experiments were performed with four conditions, each with three technical replicates, for 36 LC‐MS samples. Analysis of CD4^+^ T‐cell lysates for the three experiments identified 6997, 7807, and 6353 peptides in each independent experiment with 4496 peptides observed across all experimental conditions (Table S1, Supporting Information). For statistical purposes (Figure 1B), samples from non‐ManLAM‐treated (i.e., untreated) resting CD4^+^ T cells served as base value or control, and comparisons were made between following groups: Group (1) activated CD4^+^ T cells; Group (2) activated CD4^+^ T‐cells + ManLAM; and Group (3) resting CD4^+^ T cells + ManLAM. We also compared the peptide abundances for: Group (4) activated CD4^+^ T cells + ManLAM compared to activated CD4^+^ T cells that were not exposed to ManLAM. Group 4 contained the key comparison to determine the effect of ManLAM on the proteome of activated CD4^+^ T cells. Likelihood ratio test based on the mixed‐effect model was applied to identify potential winners in statistical testing. The final winners were based on FDR‐adjusted *p*‐value cut‐off of 0.1. The number of potential winners (uniquely identified differentially abundant peptides) and differentially expressed proteins for these four experimental groups from the three experiments are listed in Figure 1B. Overall, 178, 95, 33, and 149 peptides were identified representing 134, 70, 29, and 131 differentially expressed proteins in comparison Groups 1–4, respectively. These peptides are listed in Table S2A–D, Supporting Information. The overlap and distribution of differentially expressed proteins for Groups 1–3 are shown in the Venn diagram in Figure 1C.

ManLAM treatment induced a minor change in the resting CD4^+^ T‐cell proteome (Group 3) with changes in 29 proteins. As expected, activation of CD4^+^ T cells resulted in a much larger change with 134 proteins altered (Group 1). ManLAM treatment reduced this change in the activated CD4^+^ T‐cell proteome (Group 2) to 70 proteins, consistent with its attenuating effects on T‐cell activation.

We next analyzed the effect of ManLAM on the proteome of activated CD4^+^ T cells (Group 4), the key focus of these proteomic experiments. One hundred thirty‐one proteins were significantly altered in abundance between activated CD4^+^ T cells exposed or not exposed to ManLAM before activation (Figure 1B). A list of these 131 proteins is found in Table S2D, Supporting Information. Overall, quantitative label‐free MS analysis revealed a global profile of the impact of ManLAM on the activated CD4^+^ T cells proteome. Group 4 formed the major focus of subsequent analyses, since we wished to determine how ManLAM affected the activated CD4^+^T‐cell proteome.

### 3.2 Protein–Protein Interaction Networks Affected by Manlam in Activated Cd4^+^ T Cells

**Figure 2:**
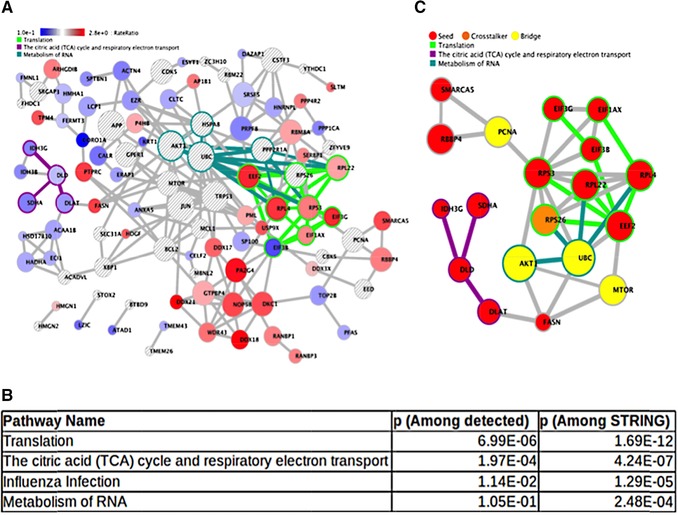
Protein–protein interaction (PPI) network affected by ManLAM in activated CD4^+^ T cells. A) Crosstalker subnetwork of proteins created using STRING as source PPI. Crosstalker designates each node with a distinct color on the basis of rate ratio between the activated versus activated + ManLAM‐treated CD4^+^ T cells (Group 4) selected by the Crosstalker algorithm (YourOmics, Inc.; www.youromics.com). B) Enriched pathway modules in the Crosstalker network are listed based on their corresponding *p*‐value. Network‐enriched pathways are overrepresented in the nodes of a single Crosstalker network as determined by a Fisher's exact test. C) Three of the top most functionally enriched modules (translation, TCA cycle, RNA metabolism) were connected using the Find Path feature of Crosstalker to build a subnetwork showing PCNA, Akt, and mTOR acting as bridges between the cluster modules. Green edges represent the connectivity among the molecules in each module. Seeds (in Red) were present in the input list and qualified as significant by the Crosstalker algorithm. Bridge (Yellow) and Crosstalker (Orange) proteins are nonseed proteins recruited because they are significant in the network (Crosstalker nodes) or connect network modules by shortest paths (bridge nodes).

Next, we determined the major protein networks dysregulated by ManLAM in activated CD4^+^ T cells.^31^ PPI maps are useful for understanding relationships among proteins important for metabolism or signaling, and clusters or modules within protein networks are dysregulated in important cellular processes or in diseases.^32^ We used Crosstalker to assess the topological connections and map subnetwork modules using all 131 proteins (e.g., seeds) identified as significantly affected by ManLAM in activated CD4^+^ T cells. We explored their relationships in the context of a well‐known PPI network, in this case from STRING, returning only those genes or proteins that have a demonstrated “closeness” (as reflected by the Crosstalk topology score). Using the above 131 proteins as seeds, Crosstalker returned a subnetwork where 107 of the seeds were both mapped to STRING and found to be significantly connected by the algorithm (Figure 2A). To incorporate novel but topologically important nodes, the algorithm adds Crosstalkers to the networks (orange nodes in Figure 2C). Eleven novel Crosstalker nodes were also added that showed significant proximity to the 107 seed nodes. Bridge nodes (yellow nodes in Figure 2C) were included in the visualization to connect subnetwork modules with a shortest path. In our analyzed datasets for Group 4, Crosstalker deduced a subnetwork comprised of 69 seeds plus 11 Crosstalkers connected by bridge nodes as shown based on RR of the seed proteins (Figure 2A).

These networks and their component proteins were assessed for pathway enrichment, and pathway and network elements were simultaneously visualized. Using Reactome and Pathway interaction database pathway sets, we identified the top pathways associated with the Crosstalker networks listed in Figure 2B. Notable pathways include protein translation, citric acid cycle, and respiratory complex and RNA metabolism. The network workspace that includes the above results can be visualized and explored with a web browser using the link supplied in Figure 2 legends. In this way, specific pathway/network connections can be explored in detail to further understand the data.

We also explored a subnetwork where top‐enriched pathways and associated molecules (such as Translation [Eef2, Rpl4, Eif3g, Rpl22]; TCA cycle [Dlat, Dld, Idh3h, Sdha]; and RNA metabolism) were interconnected densely. We used the Path Finder feature from the Crosstalker menu bar to highlight the functional association within the full result network (Figure 2A) of nodes in the translation and TCA cycle pathways as well as PCNA. Figure 2C visualizes paths connecting and built through bridges PCNA, UBC, Akt1, and mTOR identified by Crosstalker. These bridge nodes are part of different regulatory pathways such as regulation of cell cycle (PCNA), metabolic processes (mTOR), and ubiquitination (UBC). These protein clusters shown to be centrally connected through Akt and mTOR in ManLAM‐treated cells are shown in the subnetwork.

Overall, the network analysis provided a unified framework to assess the dysregulated protein sets in activated CD4^+^ T cells exposed to ManLAM (Group 4) and connected them to key signaling molecules of interest. The workspace can be used to further explore these connections.

### 3.3 ManLAM Inhibits PCNA, A Regulator of Cell Cycle Progression in Activated CD4^+^ T Cells

**Figure 3:**
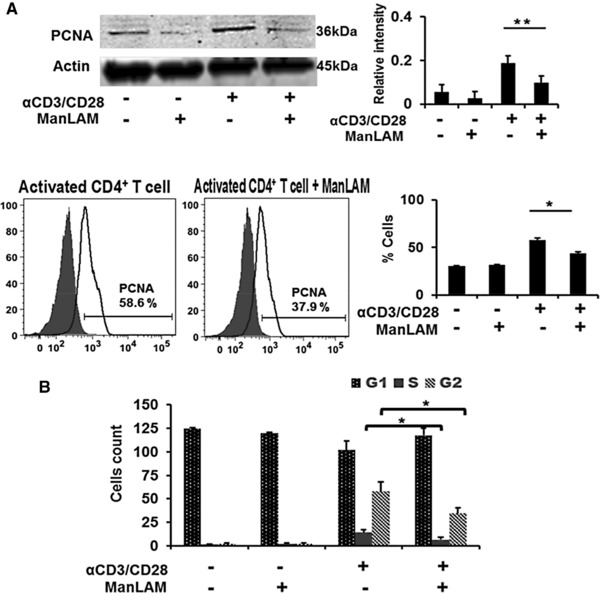
ManLAM inhibits PCNA, a regulator of cell cycle in activated CD4^+^ T cells. A) WB and intracellular staining (ICS) for PCNA expression in CD4^+^ T cells in a representative experiment. Densitometry results are the mean ±SD of the ratio of PCNA/actin for three experiments. Summary of three ICS experiments is expressed as mean fluorescence intensity activated CD4^+^ T cells with and without ManLAM pretreatment. B) Cell cycle analysis of the effect of ManLAM on resting and activated CD4^+^ T cells as measured by propidium iodine (PI) staining followed by flow cytometry. Results of a representative experiment of three are shown.

We next sought to validate independently the significance and directionality of changes in protein abundances identified by the combined MS and network analysis as potentially dysregulated by ManLAM. We first focused on PCNA, a key protein identified as a bridge node in the subnetwork analysis (Figure 2A and C). Mixed‐effect model‐based statistical analysis identified a unique peptide of PCNA significantly differentially expressed (RR 0.417 and *p*‐value 0.0924) in activated CD4^+^ T cells compared to resting CD4^+^ T cells. While MS detected PCNA upregulation in activated CD4^+^ T cells, PCNA peptides did not meet our statistical criterion for change, that is, presence in all three experiments, when we compared CD4^+^ T cells activated in the presence or absence ± ManLAM (i.e., Group 4). Proteomics did indicate p27 (i.e., CDkn1b), a regulator of cell cycle progression, was upregulated by ManLAM. Increased p27 expression is directly associated with decreased PCNA expression, a cell proliferation marker.^33^ WB demonstrated increased PCNA expression in activated CD4^+^ T cells confirming MS‐based peptide analysis that was decreased by ManLAM. This result supported PCNA's identification as connecting node in T‐cell proliferation and associated functions (Figure 2B). Intracellular staining for PCNA confirmed the WB results (Figure 3A). In light of these WB and functional results, we returned to our proteomic results and found using more relaxed criteria, that is, presence in two of three experiments, five PCNA peptides in activated CD4^+^ T cells and these were all significantly reduced in the presence of ManLAM.

To determine whether inhibition of PCNA affected cell cycle progression and proliferation, PI staining was used to measure DNA content as indicator of the different stages of cell division. More cells were static at SG2M in activated CD4^+^ T cells treated with ManLAM compared to untreated activated cells. ManLAM pretreatment had no effect on PI staining patterns of resting CD4^+^ T cells (Figure 3B, bar graph). These results provide independent confirmation that ManLAM dysregulates PCNA, leading to inhibition of cell cycle progression.

### 3.4 ManLAM Inhibits Akt and mTOR Phosphorylation in Activated CD4^+^ T Cells

**Figure 4:**
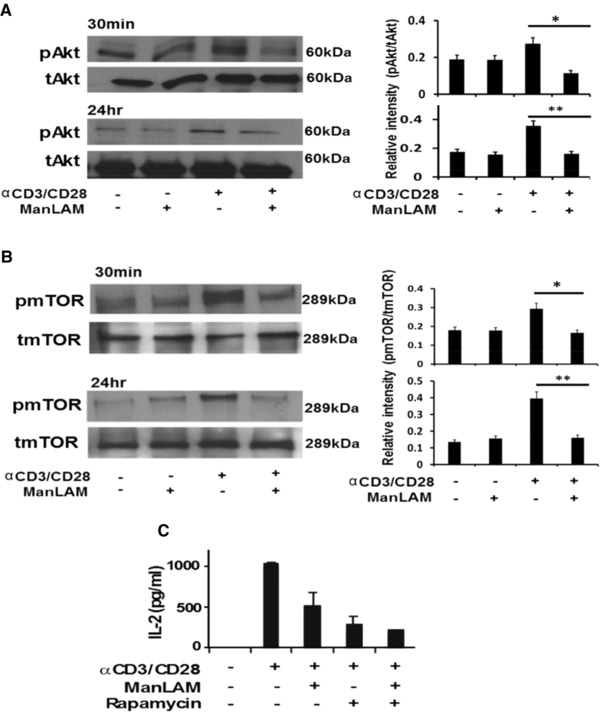
ManLAM inhibits Akt and mTOR phosphorylation in activated CD4^+^ T cells. A) Total (tAkt) and Ser473 phosphorylated Akt (pAkt) expression measured by WB 30 min and 24 h in lysates of CD4^+^ T cells cultured with and without anti‐CD3/CD28 in the absence or presence of ManLAM. Expression of pAkt was quantitated by densitometry and expressed as ratio of pAkt to tAkt. WB shown is representative of one experiment. Densitometry is based on three separate experiments. B) Total (tmTOR) and Ser2448 phosphorylated mTOR (pmTOR) expression measured by WB 30 min and 24 h in lysates of CD4^+^ T cells cultured with and without anti‐CD3/CD28 in the absence or presence of ManLAM. Expression of phosphorylated mTOR was quantitated by densitometry and expressed as a ratio of pmTOR to tmTOR. WB shown is representative of one experiment. Densitometry results are based on three separate experiments. C) Effect of 1 h of ManLAM (40 μg/mL) or rapamycin (10 nM) pretreatment on IL‐2 production by anti‐CD3/CD28 activated CD4^+^ T cells as measured by IL‐2 ELISA. Results shown represent the mean ± SD of three experiments.

In the Path Finder shortest pathway analysis, that is, selecting nodes from each module to show possible proximal connectivity measured as a path among selected nodes, Akt and mTOR act as central bridge molecules that link the three top protein networks dysregulated by ManLAM (Figure 2B). Akt and its downstream partner mTOR can be activated through TCR signaling alone, but this activation is enhanced and prolonged by CD28 signaling by promoting PI3K signaling. Upon activation by CD28 signaling, T cells enter into a proliferative state through IL‐2 cytokine gene expression and secretion as the result of coordinating different T‐cell transcription factors, including NFκB.^34^, ^35^ Akt and mTOR function are regulated by phosphorylation and not necessarily through changes in levels of protein expression, and thus may not be detected as differentially abundant in label‐free MS experiments. To determine if Akt and mTOR were regulated by ManLAM, lysates of CD4^+^ T cells were analyzed by WB for total and phosphorylated Akt and mTOR. Decreased phosphorylation of both molecules was observed both at 30 min and 24 h in activated CD4^+^ T cells exposed to ManLAM compared to activated CD4^+^ T cells (Figure 4A and B) not exposed to ManLAM.

Rapamycin is an inhibitor of mTOR, and inhibits T‐cell activation and cytokine secretion.^36^ We compared ManLAM to rapamycin for its ability to inhibit CD4^+^ T‐cell activation. Resting CD4^+^ T cells were pretreated with either rapamycin (10 nM) or ManLAM for 1 h at 37 °C followed by activation with anti‐CD3/CD28 for 24 h. Rapamycin inhibited IL‐2 secretion by 80% with ManLAM coming close at 50–60% (Figure 4C). These findings provide additional evidence that ManLAM inhibits mTOR in activated CD4^+^ T cells. Given the central role of this pathway in the different protein networks dysregulated by ManLAM, this suggests that interference with Akt‐mTOR signaling is the major downstream pathway affected by ManLAM in activated CD4^+^ T cells.

### 3.5 ManLAM Inhibits Expression of Otub1 and Usp9x, and Also NF‐κB in Activated CD4^+^ T Cells

**Figure 5:**
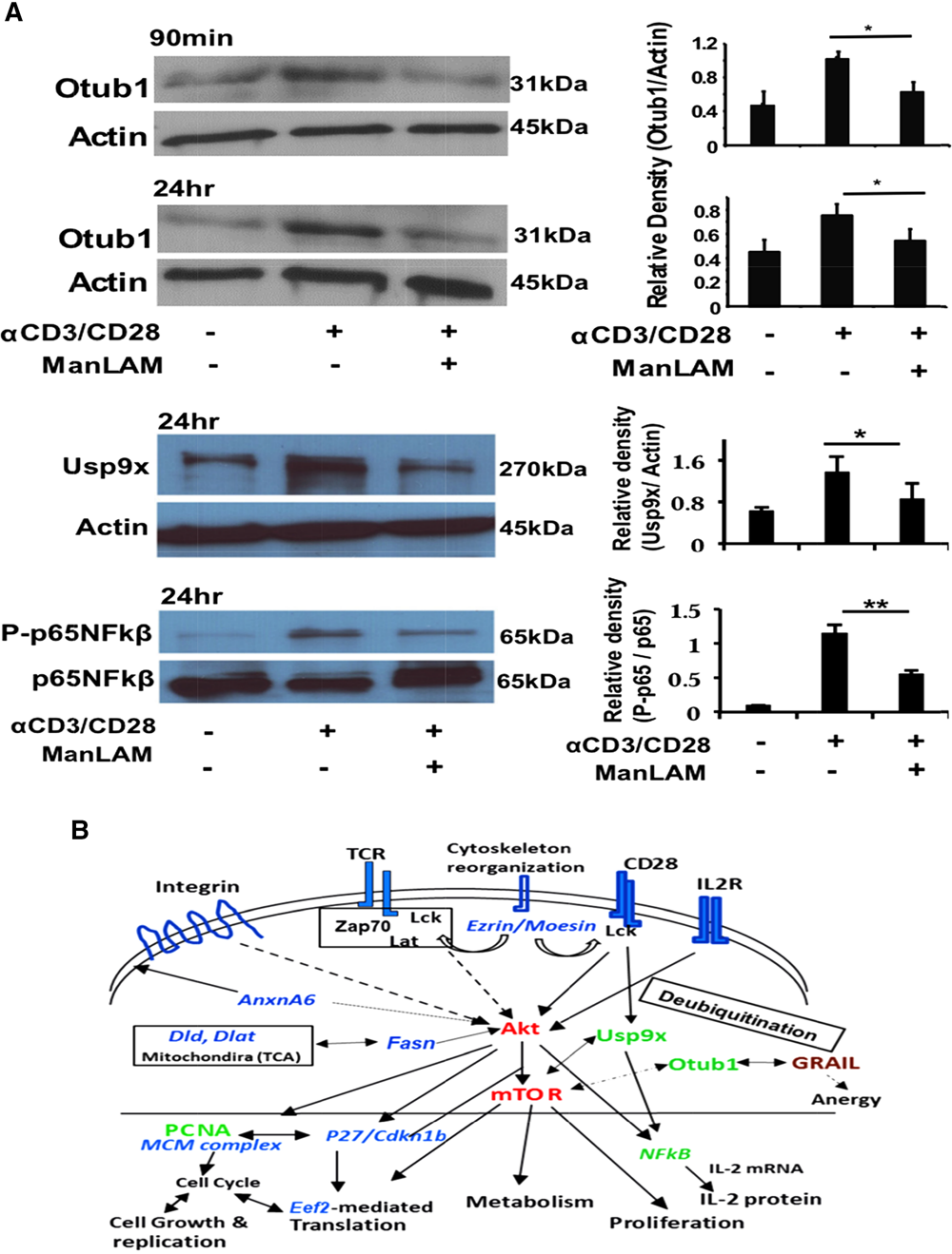
ManLAM inhibits expression of deubiquitinase enzymes otubain 1 (Otub1) and Usp9x and Usp9x's regulation of NF‐κB in activated CD4^+^ T cells. A) Deubiquiting enzymes Otub1 and Usp9x expression were measured by WB in lysates of CD4^+^ T cells before and after activation by anti‐CD3/CD28 in the absence and presence of ManLAM. Phospho‐p65 NF‐κB expression was also measured in lysates from activated T cells. Expression of Otub1 and Usp9x were quantitated by densitometry and expressed as a ratio relative to actin. P‐p65 NFκB was quantitated by densitometry and expressed as a ratio relative to total p65NFκβ. WB shown is representative of one experiment. Densitometry results are based on three separate experiments. B) Model of ManLAM's effect on activated CD4^+^ T‐cell signaling and function. ManLAM's global inhibition of proximal TCR signaling (Lck, Zap70, LAT) and CD28 signaling results in inhibition of Akt and mTOR phosphorylation, and Akt‐mTOR‐regulated processes, including protein synthesis, cell growth and differentiation, and metabolism. Akt regulates IL‐2 production. In addition, mTOR regulates Otub1, a deubiquinating enzyme, that binds and regulates GRAIL, a protein that regulates T‐cell anergy. Proteomics also revealed that ManLAM affects TCR and CD28 signaling mediated ubiquitination processes, and provided links between Usp9x, mTOR, and NF‐κB. In this diagram, some identified proteins were listed to show ManLAM's effect on the T‐cell proteome. Intracellular proteins in blue were identified by proteomics and downregulated by ManLAM. Proteins in green (PCNA, Otub1, Usp9x) were identified by proteomics and ManLAM‐induced changes validated by WB. Proteins in red (Akt, mTOR) were identified by network analysis and inhibition of their phosphorylation validated by WB.

ManLAM‐induced inhibition of proximal TCR signaling results in CD4^+^ T‐cell anergy by upregulating GRAIL (gene related to anergy in lymphocytes) protein.^8^, ^11^ GRAIL is an E3 ubiquitin ligase. MS did not identify GRAIL but proteins regulating ubiquitination were identified by mixed‐effect model analysis. As shown in the network modules, one such protein, Usp9x (RR = 2.0453 and *p*‐value = 0.0658), a deubiquitinating enzyme, was differentially regulated by ManLAM (Table S2D, Supporting Information). Usp9x interacts with bridge nodes mTOR and UBC (ubiquitin C) by network analysis (Figure 2A). Another deubiquitinating enzyme, Otub1, directly interacts with GRAIL and also is regulated by mTOR.^37^, ^38^ MS analysis identified Otub1peptide among the 4496 peptides across all experiments but Otub1 did not qualify as differentially abundant in our statistical analysis. Usp9x regulates mTOR activity by interacting with the MALT1/BCL10/Carma1 complex that is responsible for NF‐κB activation triggered by CD28 signaling.^39^, ^40^ To determine if both Otub1 and Usp9x were regulated by ManLAM, Otub1, and Usp9x, protein expression were analyzed by WB. WB analysis revealed that after anti‐CD3/CD28 stimulation, Otub1 was increased after 90 min and further by 24 h, and Usp9x at 24 h (Figure 5A). Pretreatment with ManLAM resulted in inhibition of Otub1 and Usp9x at 24 h. ManLAM also decreased phosphorylation of NF‐κB as determined by WB (Figure 5A) extending the finding of reduced Usp9x in ManLAM‐treated activated CD4^+^ T cells. These validation experiments indicate that ManLAM affects CD28 signaling by inhibiting Usp9x‐mediated regulation of mTOR and NF‐κB. Thus, apart from interfering with proximal TCR signaling (signal 1), ManLAM also inhibits CD28 signaling (signal 2), resulting in a dominant effect on mTOR phosphorylation and NF‐κB activation via Usp9x. In parallel, ManLAM's effect on another deubiquitinase enzyme Otub1 provides a link between mTOR and GRAIL‐mediated CD4^+^ T‐cell anergy.

## 4 Discussion

Exposure of CD4^+^ T cells to Mtb cell wall glycolipid ManLAM in the proteome of CD4^+^ T cells before activation through TCR‐CD3 and CD28 results in marked changes in the T‐cell proteome. TCR‐CD3 signaling starts with phosphorylation of Lck, Zap70, and LAT, and formation of a complex LAT signalosome with SLP‐76, involving at least 90 proteins and resulting in the activation of multiple serine–threonine kinases.^41^, ^42^ These serine–threonine kinases include the PKC family, Raf1, Erk1/2, and Akt, and further activate multiple downstream pathways.^42^ Optimal T‐cell activation and proliferation requires participation of CD28 (Signal 2) and its signaling cascade, aided by the interaction of IL‐2 with IL‐2R.^43^ In this study, we used proteomics and systems biology to dissect the major impact of ManLAM on the complex pathways that follow signaling through TCR‐CD3, CD28, and IL‐2R. Our results indicate that ManLAM in addition to affecting proximal TCR‐CD3 signaling also impacts CD28 signaling resulting in the Akt‐mTOR pathway as the primary signaling pathway affected by ManLAM in activated CD4^+^ T cells.

A number of studies have reported changes in the T‐cell proteome induced by activation through the TCR–CD3 complex and/or through CD28, but have focused primarily on proximal signaling events and used older gel‐based 2D electrophoresis.^12^, ^13^, ^14^, ^15^, ^44^, ^45^ Earlier studies also focused on aspects of the proteome associated with the lipid membrane modifications involved in early T‐cell signaling. As expected, there is substantial overlap in the proteome of activated CD4^+^ T cells in our studies compared to these earlier studies. Differences in our activated CD4^+^ T‐cell proteome compared to earlier studies are due to differences in technology and analytic strategy resulting in a larger number of identified proteins changed upon TCR/CD3 and CD28 activation.

Numerous approaches have been developed to identify biological modules and clustering interactions.^46^, ^47^, ^48^ Network analysis with Crosstalker determined the relationships between 131 differentially regulated proteins, identified the major functional networks, and enriched modules representing pathways affected by ManLAM in activated CD4^+^ T cells. Cross‐talker identified Akt and mTOR as central bridge molecules that linked molecules in the top two enriched pathways dysregulated by ManLAM, that is, translation and the TCA cycle. The proteomic data indicated that CD4^+^ T cells exhibited signs of hyporesponsiveness in the presence of ManLAM, reflected in decreased cellular proliferation with cell cycle arrest in SG2M, and decreased PCNA and increased p27 expression.

Akt and mTOR were the core proteins connecting the different clusters of molecules in deduced subnetworks, and ManLAM inhibited their activation. Akt and mTOR are well‐characterized downstream effector molecules that connect the TCR–CD3, CD28, and IL‐2R signaling for activation, proliferation, and IL‐2 gene transcription and translation in T cells.^49^ mTOR's role in T‐cell activation and its regulation by rapamycin has been studied functionally and by proteomics in light of rapamycin's role in preventing rejection of transplanted organs.^19^, ^50^ Since ManLAM inhibited mTOR and CD4^+^ T‐cell activation, it was not surprising that there was overlap in the dysregulated proteomes of ManLAM and rapamycin‐treated CD4^+^ T cells.

Ubiquitination‐mediated degradation is a major means to regulate protein function. We found that de‐ubiquitinating enzyme (DUB) Usp9x was dysregulated in the presence of ManLAM. Usp9x regulates proximal TCR–CD3 signaling molecules and helps maintain a state of T‐cell self‐tolerance.^51^ Usp9x also upregulates and stabilizes the Carma1/BCL10/MALT1 (CBM) complex responsible for MAP‐kinase and NF‐κB activation. Optimal Carma1/BCL10/MALT1 activation requires signaling both through TCR–CD3 and CD28.^39^ Usp9x also upregulates mTOR function by stabilizing its interaction with MALT1/Carma1, and thus provides a proteomic link to the Akt‐mTOR pathway.^40^ We confirmed Usp9x's inhibition by ManLAM by WB and found decreased NF‐κB activation in ManLAM activated CD4^+^ T cells.

Another DUB, Otub1 interacts with GRAIL and regulates the delicate balance between immune activation and anergy.^38^ WB analysis revealed decreased Otub1 expression in ManLAM‐treated activated CD4^+^ T cells. Otub1 forms a trimolecular complex with USP8 and GRAIL, and upregulated Otub1 promotes GRAIL's proteosomal degradation. Activated mTOR upregulates Otub1 stabilizing the trimolecular complex allowing GRAIL's degradation and thus T‐cell proliferation to proceed. Inhibition of mTOR phosphorylation by ManLAM suppresses Otub1 expression and thus persistence of GRAIL. Independent studies from our group have determined that ManLAM increases GRAIL expression and induces functional anergy in CD4^+^ T cells.^11^

Proteomic analysis also demonstrated an effect of ManLAM on cytoskeletal rearrangement linking upstream signaling events with the downstream effects on Usp9x and Akt‐mTOR. MS and network analyses found decreased Ezrin (Ezr) and Moesin (Msn), members of the ezrin–radixin–moesin complex, expression in the presence of ManLAM. Ezrin is phosphorylated by Lck during the earliest phase of T‐cell activation.^52^ Phosphorylated Ezrin mobilizes Zap70 to CD3's ITAMs for phosphorylation by Lck and for T‐cell activation to proceed. Inhibition of Akt‐mTOR phosphorylation by ManLAM may be due to its effect on T‐cell cytoskeletal rearrangement since both Ezrin and Moesin are linked to the Akt‐mTOR axis for controlling T‐cell activation through TCR‐CD3, CD28, and IL‐2R.^53^

A recent report correlated increased HIV replication with decreased PCNA, Otub1, and dysregulation of the Akt‐mTOR pathway in infected CD4^+^ T cells.^54^ Mtb‐HIV coinfected individuals are at high risk of developing TB.^55^ Finding similar changes in the CD4^+^ T‐cell proteome by two different pathogens supports the importance of the Akt‐mTOR signaling pathway in antimicrobial defenses and its vulnerability to microbial attack. mTOR is a major regulator of autophagy and CD4^+^ T‐cell autophagy may be an additional mechanism by which pathogens can interfere with T‐cell function.^56^, ^57^

Overall, these proteomic findings combined with our functional studies in human CD4^+^ T cells and in vivo studies in murine Mtb infection support a model in which ManLAM as Mtb bacterial membrane vesicles can travel beyond the immediate environment of infected cells, that is, the granuloma, to interact with CD4^+^ T cells and likely others.^6^, ^7^, ^8^ For CD4^+^ T cells, this interaction results in inhibition of TCR–CD3 and CD28 signaling with a downstream effect on the Akt‐mTOR signaling pathway, resulting in T‐cell inhibition and anergy. This may be a mechanism for Mtb to persist despite an extensive T‐cell response reflected in a positive tuberculin skin test and strong IFN‐γ response to Mtb proteins in most infected persons. Clinical studies in humans and additional animal studies will determine the in vivo significance of this T‐cell immune evasion strategy.

**Abbreviations**
FDR: false discovery rate
GRAIL: gene related to anergy in lymphocytes
IPI: international protein index
ManLAM: mannose‐capped lipoarabinomannan
Mtb: *Mycobacterium tuberculosis*
mTOR: mammalian target of rapamycin
NF‐κB: nuclear factor kappa light‐chain‐enhancer of activated B cells
Otub1: ovarian tumor deubiquitinase ubiquitin aldehyde binding 1
PCNA: proliferating cellular nuclear antigen
PPI: protein–protein interaction
RR: rate ratio
Usp9x: ubiquitin‐specific peptidase 9, X‐linked
WB: western blot

## Conflict of Interest

The software Crosstalker is licensed from Case Western Reserve University to Neo Proteomics, Inc. (www.neoproteomics.net) and is distributed by Your Omics, Inc. (www.youromics.com). Crosstalker is freely available for noncommercial use. Mark Chance and Sean Maxwell declare a conflict of interest as they are shareholders of Your Omics, Inc.

## Supporting information

Supporting Information

Supporting Information

Supporting Information

Supporting Information

Supporting Information
